# Insights into the Alteration of Osteoblast Mechanical Properties upon Adhesion on Chitosan

**DOI:** 10.1155/2014/740726

**Published:** 2014-05-29

**Authors:** Antonia G. Moutzouri, George M. Athanassiou

**Affiliations:** Laboratory of Biomechanics and Biomedical Engineering, Department of Mechanical Engineering and Aeronautics, University of Patras, 26504 Rio, Patras, Greece

## Abstract

Cell adhesion on substrates is accompanied by significant changes in shape and cytoskeleton organization, which affect subsequent cellular and tissue responses, determining the long-term success of an implant. Alterations in osteoblast stiffness upon adhesion on orthopaedic implants with different surface chemical composition and topography are, thus, of central interest in the field of bone implant research. This work aimed to study the mechanical response of osteoblasts upon adhesion on chitosan-coated glass surfaces and to investigate possible correlations with the level of adhesion, spreading, and cytoskeleton reorganization. Using the micropipette aspiration technique, the osteoblast elastic modulus was found higher on chitosan-coated than on uncoated control substrates, and it was found to increase in the course of spreading for both substrates. The cell-surface contact area was measured throughout several time points of adhesion to quantify cell spreading kinetics. Significant differences were found between chitosan and control surfaces regarding the response of cell spreading, while both groups displayed a sigmoidal kinetical behavior with an initially elevated spreading rate which stabilizes in the second hour of attachment. Actin filament structural changes were confirmed after observation with confocal microscope. Biomaterial surface modification can enhance osteoblast mechanical response and induce favorable structural organization for the implant integration.

## 1. Introduction

Cell adhesion to surfaces is a key regulator of prominent biological processes. The adherent cell undergoes significant shape changes, including the initial membrane deformation as well as the extensive cytoskeleton reorganization [1, 2]. This process is advanced by both the cell ability to supply excess membrane and the plethora of biomaterial surface induced stimuli, allowing the cell to cope with substantial morphological changes. Thus, the success of biomaterials is determined by the initial phase of adhesive interactions between the cells and the surface of the implant. During the initial adhesion step, profound changes occur in membrane mechanical properties, which are of critical importance in implant integration, as the cell can accommodate the challenges of spreading and migration [3–5].

In bone tissue engineering research, the design of chemically modified surfaces allows the development of functional biomaterials, since it has been referred to the fact that biological tissues interact mainly with the outermost atomic layers being generally about 0.1–1 nm. Osteoblasts that have to integrate the orthopaedic implant are in direct contact with its surface, and the long-term acceptance of the material mostly relies on the initial adhesion phase and the subsequent mechanical response upon adhesion [6]. Cells have the remarkable ability to sense the rigidity of their environment, which modulates fundamental cell functions, including focal adhesion formation, spreading, and cell differentiation [7]. Alterations in the shape and the cytoskeleton upon adhesion to different substrates are probably accompanied by alterations in the mechanical properties of the osteoblasts, since the main components of cytoskeleton, as F-actin and the microtubule cytoskeleton, have been found to contribute to the mechanical stiffness of the cell [8].

Correlations between extracellular matrix (ECM) characteristics and cell mechanical properties have been of great research interest aiming at understanding how these physical and mechanical alterations affect cellular behavior. The force-deformation responses provide insight into similarities and differences in cytoskeletal microstructure between cells adhered to different substrates and in different time points of the adhesion process. The elastic moduli, viscosity, and other mechanical and rheological parameters are measures which may determine changes of the cell mechanical and rheological properties under adhesion stress and may reflect their dependence on the ECM properties [9].

A variety of methods have been developed to quantify cellular mechanical properties, such as viscoelasticity and deformability, mainly at the single cell level of analysis. These approaches include micropipette aspiration [10], atomic force microscopy (AFM) [11], cytoindentation [12], magnetic bead rheometry [13], and optical tweezers [14]. Micropipette aspiration has been one of the most accurate and pioneering methods used for studying individual cellular mechanics as it produces cell deformations, upon aspiration, by extending cell surface into the pipette. This method is utilized for applying mechanical tests on individual cells and measuring the elastic or viscoelastic properties of the whole cell, cell membrane, and various subcellular components [15]. It has been used on numerous cell types and has applied a variety of modeling techniques [10].

During the last years, the biological phenomena occurring after the first minutes of cell to surface contact were subjected to thorough examination and much effort was done to apply biophysical or biomechanical approaches to the analysis of the cell-substrate interface and to interrogate emerging binding events. There are limited studies through examining osteoblast mechanical properties on different ECM substrates or through examining osteoblast response to mechanical stimuli when adhered to different substrates.

Chitosan is a high molecular weight, linear polycationic heteropolysaccharide, consisting of N-acetyl-glucosamine and D-glucosamine. It is produced commercially by deacetylation of chitin, which is the structural element in the exoskeleton of crustaceans, giving chitosans with varying degree of deacetylation (DD). Due to its biocompatibility, biodegradability, and nontoxic properties, it has become a promising biomaterial for a wide range of biomedical applications. It has been accepted in the biomaterials field as a structural analogue of glycosaminoglycans (GAGs). Thus, chitosan may be able to mimic certain biological activities of GAGs, including binding with growth factors and adhesion proteins [16]. Protein adsorption is a crucial event of the initial step of attachment and spreading of anchorage dependent cells and further affects subsequent cellular and tissue responses. Chitosan has been used to modify the surface properties for enhancing the attachment of osteoblasts [17, 18]. It has been suggested to promote cell attachment and support the formation of the natural ECM, enhancing bone regeneration.

While morphological changes during single cell adhesion and spreading are well characterized, the accompanying alterations in cellular mechanics are scarcely addressed. In this study, the mechanical properties of osteoblasts were determined; during the adhesion procedure on chitosan and alterations of the mechanical measures with the cell spreading rate was correlated. The purpose of the work was to better understand the way in which chitosan affects these cellular responses so as to shed light onto the design and development of implants that would create such a microenvironment to provoke coordinated molecular and mechanical activities.

## 2. Materials and Methods

### 2.1. Cell Culture

For the purpose of this study, human bone marrow stromal cells were obtained by aspiration from the femoral diaphysis of patients aged 50–70 years old who underwent total or elective hip replacement. All donors have been preoperatively controlled for systematic and local infection and malignancy; specimens taken intraoperatively were sent for histology and cultures for bacteria. Approval was obtained from the Ethical Committee of the University of Patras and the study was carried out in accordance with the Helsinki Declaration. From each donor, a “single cell” suspension was prepared by repeatedly aspirating the cells successively through 19- and 21-gauge needles. The cell suspension was cultured, as described elsewhere [19], until confluence in medium containing a minimal essential medium (MEM) + 10% fetal bovine serum + 2 mM L-glutamine + 50 lg/mL L-ascorbic acid + 10 mM Na-b-glycerophosphate + 1028 M dexamethasone + 50 lg/mL gentamycin + 2.5 lg/mL amphotericin b. Cultures were incubated at 37°C in a humidified atmosphere of 95% air and 5% CO_2_. These specific conditions “direct” the bone marrow cell culture to form osteoblasts. Positive identification of the cultured cells as osteoblasts was by staining for alkaline phosphatase activity. A trypsin concentration as low as possible was used to minimize effects on cell-surface properties but still produces cell detachment in reasonable times.

### 2.2. Preparation of Chitosan Surfaces

Cover glasses were cleaned in piranha solution (30% (v/v) of hydrogen peroxide and 70% (v/v) of concentrated sulfuric acid) for 1 h, rinsed thoroughly with 18.2 MΩ water, dried in air, and heated in vacuum oven at 120°C for 1 h. Then the cleaned substrates were dipped into 1% (v/v) APTES solution in ethanol/water (95% : 5% by volume) for 10 min and washed with pure ethanol three times. After drying in a stream of nitrogen, the silanized surfaces were heated in vacuum oven at 120°C for 1 h in order to cross-link the silanized layer on the glass surface. The silanized glasses were immersed in 1 wt% glutaraldehyde (GA) solution for 3 h at room temperature, followed by rinsing with excess 18.2 MΩ water for 24 h in order to remove free GA. The glasses were incubated in 0.4 mg/mL chitosan solution (pH 5) for 24 h at room temperature. The specific chitosan used in the study was >75% deacetylated powder from crustacean shells (419419 Aldrich). The molecular weight was high (310,000–375,000 Da). The chitosan immobilized glasses were rinsed with 1.0% acetic acid solution and then rinsed with 18.2 MΩ for 24 h to remove free chitosan [20].

### 2.3. Cell Spreading

For morphological observation, osteoblasts were seeded on the control and modified surfaces at a density of 4 × 10^4^ cells/cm^2^ and examined with scanning electron microscopy (SEM). The cells attached to the surfaces were gently washed with PBS to remove nonadherent or loosely adherent cells and then fixed for 20 min with 2.5% GA in PBS. After thorough washing with PBS, the cells were dehydrated through a series of ethanol-water solutions for 20 min each, using increasing concentrations of ethanol up to 100%. The samples were then gold sputtered in vacuum and examined using a JEOL-JSM 6300 SEM microscope (accelerating voltage between 100 V and 30 kV, we worked with 20 kV, about 103 Pa specimen chamber pressure, and current about 1.5 nA). Experiments were conducted three times for each spreading time (2, 7.5, 15, 30, 45, 60, and 75 min). Five samples for each surface (glass, chitosan) were prepared in each experiment. SEM micrographs were captured digitally on three representative fields of each sample. The average cell area (*μ*m^2^) was measured using Image Pro Plus 4.01 analysis software (Media Cybernetics).

To quantify osteoblast spreading, cell-substrate contact area (subsequently referred to as “cell area”) was determined by tracing the outline of the cell at different time points using ImageJ (NIH). This method was the inverse of the one reported in recent study of deadhesion dynamics [21]. The time-dependent normalized area was quantified by dividing the difference between the cell area at time *t* and the initial spread area (i.e., *A*(*t*) − *A*_initial_) by the difference in area between the first and last time points (i.e., *A*_final_ − *A*_initial_). Thus, the plot of normalized area increases from a value of 0 (at  *t* = 0,   *A*(*t*) = *A*_initial_) to a value of 1 (at  *t* = *t*_final_, *A*(*t*) = *A*_final_). The normalized area-versus-time data were then fit to a sigmoidal curve to yield the time constants.

### 2.4. Micropipette Method

**Figure 1:**
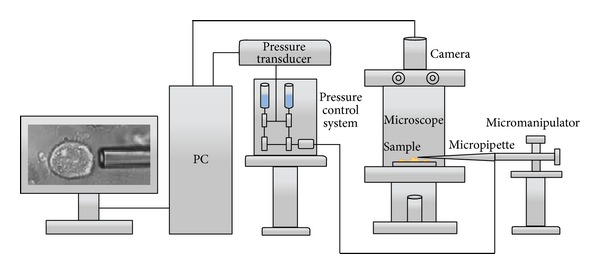
Schematic illustration of the micropipette experimental setup.

For researching the mechanical and rheological properties of the osteoblasts the elastic shear modulus, *G*, of individual cells was determined. A micropipette aspiration technique was used to aspirate the osteoblast cell membrane and obtain measurements for the applied negative pressure at each time point and the resulted aspirated length. The schematic of the experimental setup is illustrated in Figure 1.

Micropipettes were prepared by borosilicate glass tubes as described in our previous work [19, 22, 23]. The control and the chitosan samples on which the cells were attached for each of the referred studied times were placed directly onto the microscope stage. The micropipette was filled with filtered PBS-30 connected via a pressure transducer (model DP 103, Validyne, USA) to a system of varying pressure and could be moved with a 3D micromanipulator. One side of the pipette was cut at the desired internal diameter (i.d. = 4-5 *μ*m) and was directed towards a selected osteoblast. A very small negative followed by a small positive pressure (5~10 Pa) facilitated the initial manipulation of the cells so as to minimize their friction to the pipette glass surface. Pressure was increased from 100 to 700 Pa step by step very slowly to satisfy the hypothesis that the deformation (aspirated cell tongue increasing) was isothermal [24]. The tip of the micropipette and the aspirated osteoblast were observed through an inverted microscope and were also viewed and saved for further analysis with a monitoring system consisting of a camera (Pulnix TM-6CN 1/2′′ B/W CCD) and a differential pressure sensor with custom 12-bit analog to digital converter (synchronous sampling with the camera frame grabber) (“micropipette vision,” I.N. SARRIS Ltd. Company).

The membrane elastic shear modulus, *G*, was determined by the following equation: *L*/*R*_*p*_ = Φ_*p*_Δ*P*/2*πG*, where *L* is the projection length, *R*_*p*_ is the pipette radius, and *G* is the shear modulus and is related to Young's modulus *E* by *E* = 2(1 + *ν*)*G*. Φ_*p*_ is a function of the ratio of the pipette wall thickness to the pipette radius and *ν* is Poisson's ratio (*ν* = 0.45 and Φ_*p*_ = 2.0–2.1 when the ratio of the pipette wall thickness to radius is equal to 0.2–1.0) [25].

**Figure 2:**
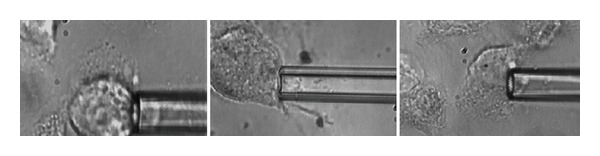
Micropipette aspiration of 3 osteoblasts at different phases of cell adhesion (30 min, 60 min, and 90 min seeding time). The radius of the pipette is 2.2–2.4 *μ*m.

Representative images of the aspirated osteoblasts in three sequenced time points of adhesion are shown in Figure 2.

### 2.5. Cell Cytoskeleton Organization

Cells were seeded onto the control and the chitosan surfaces at a density of 4 × 10^4^ cells/cm^2^ and were allowed attaching for 30 or 60 min. The samples were stained for F-actin with dye Alexa Fluor 488 phalloidin (A12379, Invitrogen) and for nucleus with DAPI (90229, Millipore) and observed with an inverted confocal microscope (ECLIPSE TE-2000U, Nikon) and 60x magnification.

### 2.6. Statistical Analysis

Statistical analysis was performed using SPSS statistical software (version 15.0, SPSS Inc.). Overall differences between groups were assessed by analysis of variance (ANOVA) and individual paired comparisons were analyzed post hoc with Scheffe's test. Results were considered to be statistically significant when *P* < 0.05. Data are expressed as mean ± standard deviation (SD).

## 3. Results

### 3.1. Cell Spreading

**Figure 3:**
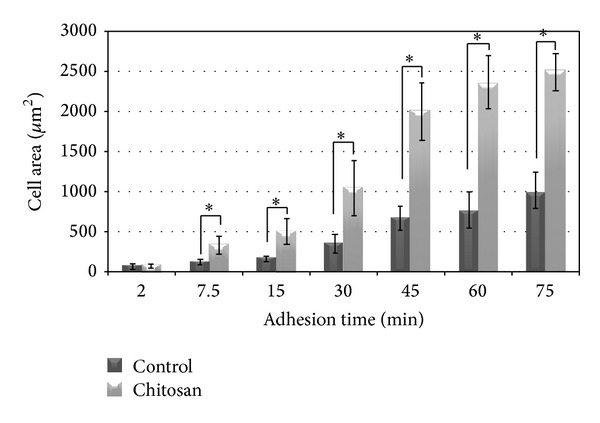
Mean ± SD values of cell area of osteoblasts attached on control and chitosan-coated surfaces after different seeding times; *P < 0.0001.

Quantitative measurement of the area of the attached cells using Image Pro software showed that the osteoblast-substrate contact area was higher when the cells were attached on the chitosan than on the control surface (Figure 3). Except for the initial time point of 2 min, where there was no statistical difference between the two groups, the mean cell area on chitosan was significantly higher (*P* < 0.0001) than on the control for all time points (7.5, 15, 30, 45, 60, and 75 min).

**Figure 4:**
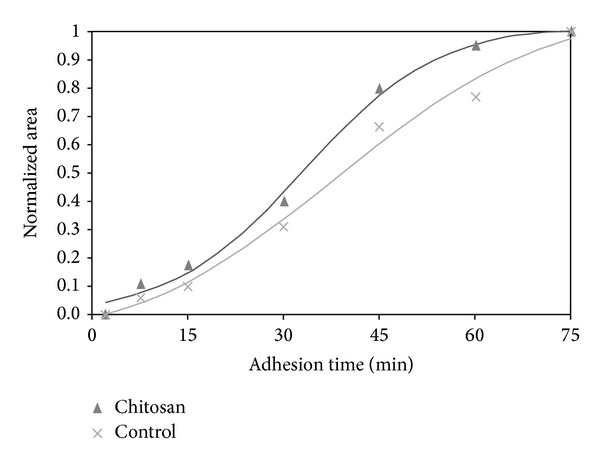
Quantification of cell shape changes during adhesion.

To quantify spreading kinetics, we plotted the normalized cell area as a function of time and fitted the experimental data with the Boltzmann equation to obtain the time constants *τ*_1_ and *τ*_2_ (Figure 4). In both cases, the adhesion response of these cells was sigmoidal and composed of three well-defined phases: the initial cell-surface attachment, a rapid spreading, and a plateau. For osteoblasts attached on glass surface, *τ*_1_ was found around 38 min, whereas on chitosan it was found around *τ*_1_~33 min, indicating a slightly faster initial response. Similarly, compared to *τ*_2_~17 min on control, *τ*_2_~10 min on chitosan corresponded to approximately 40% faster response.

### 3.2. Mechanical Properties

**Figure 5:**
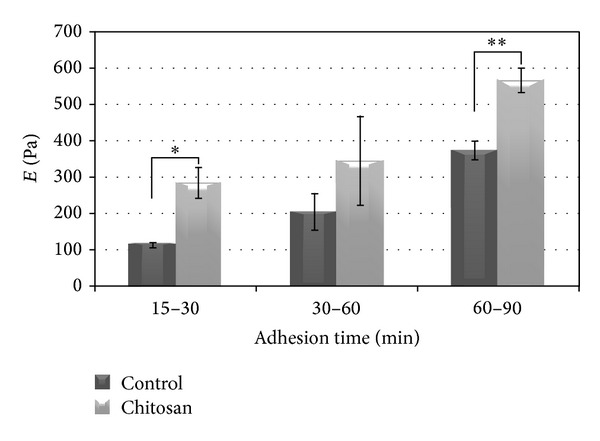
Mean ± SD values of elastic modulus of osteoblasts attached on control and chitosan- coated surfaces after different seeding times; *P < 0.05 and **P < 0.01.

Examining the changes in the mechanical properties of osteoblasts during spreading, the results showed that the elastic modulus, *E*, was found to increase in the course of cell spreading for both chitosan and control surfaces (Figure 5). The highest difference was observed for the cells over 60 minutes, exhibiting an increase by almost a factor of 2 for the control surface and more than 1.5-fold increase for the chitosan surface (*P* < 0.05 and *P* < 0.01 resp.) compared to cells allowed attaching for 30 to 60 minutes.

Differences were observed in the elastic modulus of osteoblasts attached on chitosan as compared to the cells attached on the control surface throughout all culture times (Figure 5). Specifically, for the first 30 min of cell attachment the elastic modulus *E* was found to be 150% higher for cells attached on chitosan compared to control (*P* < 0.05). Cells allowed attaching for a time between 30 and 60 minutes on chitosan again exhibited a higher value for *E* compared to the control surface, although the difference was not statistically significant (*P* = 0.137). For more than 60 minutes of attachment time, mean elastic modulus of osteoblasts on chitosan was found 52% higher, a difference which was statistically significant (*P* < 0.01).

### 3.3. Cell Cytoskeleton Organization

**Figure 6:**
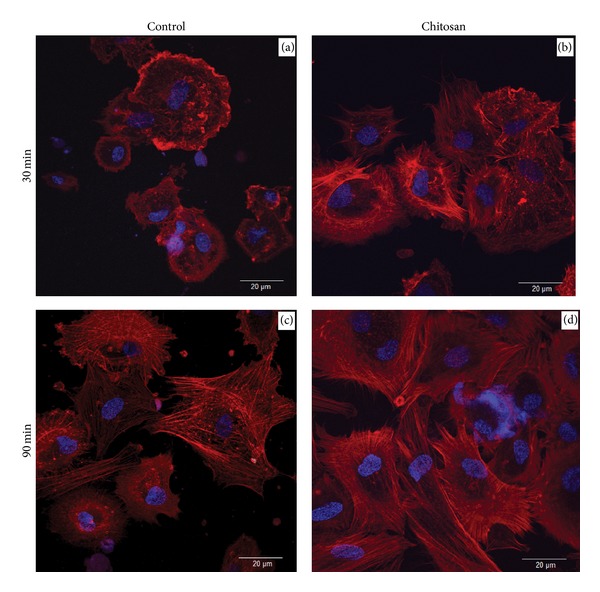
Fluorescent staining of the F-actin cytoskeleton (red) and DNA (blue), showing cell attachment and spreading after 30 and 90 minutes of cell seeding on chitosan-coated and control glass surfaces.

The imaging of the cell spreading after 30 and 90 minutes is showed in Figure 6 so as to ensure that gross changes between the substances in the initial and advanced spreading phases are captured.

The cytoskeletal organization is determined by actin labeling with phalloidin. This staining demonstrates the presence of stress fibers in the cells cultured on the samples. Osteoblasts on chitosan were significantly different in appearance, being profoundly spread and demonstrating a morphology with a well-organized actin cytoskeleton. Filopodia and lamellipodia cytoplasmic digitations were noticeably more evident on the chitosan samples for both time points tested.

## 4. Discussion

Alterations in osteoblast mechanical properties upon adhesion are likely to be an important factor in the modulation of bone cell functions and in the cell response to biomaterials. During the adhesion process, prominent cell shape changes take place which affect many cellular functions, like growth, proliferation, differentiation, and motility, all detrimental for the implant success [26]. Several studies lately support the fact that changes in the mechanical properties of cells reflect changes in cytoskeletal structure and composition and predispose for any of cell movement [5, 27, 28]. Furthermore, it has been suggested that the substrate nature, meaning substrate stiffness, nanotopography, and chemistry, affects the mechanical properties of cells, although few studies have examined the effect of surface characteristics on osteoblasts [8, 29, 30].

Chitosan, a structural analogue of GAGs, which may be able to mimic certain biological activities, such as binding with growth factors and adhesion proteins, has been used to modify the surface properties for enhancing the attachment of osteoblasts [17, 18]. It has been suggested to promote cell attachment and support the formation of the natural ECM, enhancing bone regeneration.

In this work we used the micropipette aspiration technique to measure the elastic modulus of osteoblasts on chitosan-coated and control substrates in different time points of cell adhesion. The aim was to quantify alterations in the mechanical properties during the initial phase of the cell-substrate interaction and to investigate possible correlations with the progress of adhesion and the cytoskeleton reorganization.

Our results demonstrate higher values of the elastic modulus of osteoblasts attached on chitosan-coated glass compared to the uncoated control surface, indicating that the surface characteristics indeed have an effect on cell mechanical properties. This finding is generally consistent with several works in the literature. Takai et al. [8] reported higher mean apparent elastic modulus of osteoblasts on various ECM proteins compared to osteoblasts plated on glass. The authors attributed the increase in the stiffness to the actin stress fiber formation in association with focal adhesions that form at integrin binding sites. In their study, disruption of the actin cytoskeleton resulted in a 2.5-fold decrease of apparent modulus. Hansen et al. [30] demonstrated that cells cultured on nanotopographic surfaces had a distribution of cellular modulus towards higher values relative to cells on flat control surfaces. They also found a substrate chemistry effect on the mechanical properties, however, not as great as the topography effect.

The lengthened and most flattened cells are believed to be strongly adhered to the surface, unlike the more compact and higher ones. In an attempt to establish a relationship between the local mechanical properties and the cytomorphological aspects of the cell, Simon et al. using AFM showed that the values of elastic moduli for strongly adherent cells were between 8 and 400 kPa, while for the weakly adherent cells the values varied between 0.6 and 60 kPa [27].

In our study, using a phalloidin labeling method, we demonstrated that remarkable differences appeared in the apparent morphology of the osteoblasts on the two substrates during the spreading process. The structural changes of the filaments have occurred as early as 30 minutes of cell attachment, confirming the early process of cell spreading on the chitosan nanostructured surface, compared to the glass flat control surface. This early upregulation of filopodia might be in direct correlation with the adhesive bonds formed, which would also further support our results of increased adhesion strength [19]. Moreover, it has recently been suggested that the cell is guided in the direction where the geometrical constraints allow the filopodial contacts to mature by forming a maximal number of adhesive bonds [31].

Several properties of chitosan have been reported to strongly influence cell attachment and bioactivity* in vitro*, including the DD, origin of chitin source, and the surface characteristics of the final membrane coating. Recent publication indicates that chitosan with increased DD is correlated with increased surface roughness and fibronectin. A higher DD was also shown to facilitate attachment and proliferation of cells [32]. Protein adsorption is a crucial event of the initial step of attachment and spreading of anchorage dependent cells and further affects subsequent cellular and tissue responses. Our results using high DD chitosan suggest rapid changes of the elastic modulus, *E*, during the phase of attachment implying high rate of cytoskeleton reorganization in the first 30 min and a significant increase of cell strength before the cell becomes completely spread. These results also support findings of the other investigators that osteoblastic cell attachment and growth are favored on chitosan, supporting differentiation and secretion of ECM molecules [18, 33–35].

Subtle differences in actin filament spatial reorganization have been suggested to have a substantial effect on cellular mechanical behavior. Jaasma et al. [36] found a 1.25- to 1.70-fold increase in osteoblastic cell stiffness when cells were submitted to 1 to 2 Pa shear stress compared to static cells. The applied shear stress was shown to cause changes in the organization of the actin cytoskeleton, which was attributed to play a major role in the whole cell mechanics. In another study, Stroka and Aranda-Espinoza showed that human endothelial cells increase their stiffness as they spread. They suggested this to be expected since the cells transform from round spheres with a layer of cortical actin, to spread out objects adhering to the substrate and containing a stiff network of cross-linked actin filaments and cortical actin under tension [37]. This potential increase in tension of the F-actin architecture was suggested to contribute to the overall increase in cell stiffness and is likely caused by myosin II, a motor protein known to cross-link actin filaments. Recently, Darling et al. [38] demonstrated that, under spread conditions, cells were stiffer, with the osteoblasts showing the greatest increase in average moduli (1.5-fold increase in *E*_elastic_) compared with other cell types.

Our results are in agreement with the above findings. Interestingly, there is a significant increase in filopodia projections on the control surface between 30 and 90 minutes of adhesion (Figure 6), in fact, a greater difference than that of the corresponding on the chitosan surface, implying that the absence of chitosan may only delay the cytoskeleton reorganization. This might be explained by the fact that the initial cell mechanical behavior determined by the low elastic modulus values anticipates higher rate of filopodia projection. This is in agreement with other investigators' suggestions that the elasticity of the cell membrane influences the protrusion dynamics of the filopodium [39]. So, the early spreading observed on chitosan is accompanied by an increased stiffness of the membrane, while the flexible membrane on the glass flat surface of 30 minutes enhances the rate of the filopodial growth up to 90 minutes.

It would be of special interest to further correlate these results with our previous findings on adhesion strength of osteoblasts on chitosan [19]. We have reported that the detachment strength was significantly increased (40 × 10^−7^ Nt s) within the first hour of cell attachment, where the highest rate of cell spreading was noted (2200 *μ*m^2^). The detachment process was conducted employing the micropipette aspiration technique and the whole cell detachment was completed undisturbed, meaning that the cell membrane is intact throughout all time points [19]. More specifically, the cell detached sustained its integrity at the highest applied pressure of 4000 Pa on the pipette entrance area of 12.56 × 10^−12^ m^2^, corresponding to an applied force of 5 × 10^−8^ Nt. The findings of the present study confirm the correlation of cell adhesiveness with the stiffness alterations accompanying cell spreading, since the membrane strength has an elastic shear modulus of 600 Pa, permitting the separation of the cell from the surface on which it is attached without the membrane disruption, something that would have been inevitable in the case of a softer cell material.

## 5. Conclusion

The results of the present study reveal that chitosan substrate stimulates fast osteoblast response, displaying rapid cell spreading and cytoskeleton reorganization. Cell adhesion has taken place in three distinct phases, as determined by the spreading kinetics. The osteoblast mechanical properties were significantly changed, with the cell exhibiting higher stiffness when there was a depletion of the cell excess membrane reservoir.
